# On the Stability of Exponential Backoff

**DOI:** 10.6028/jres.108.027

**Published:** 2003-08-01

**Authors:** Nah-Oak Song, Byung-Jae Kwak, Leonard E. Miller

**Affiliations:** National Institute of Standards and Technology, Gaithersburg, MD 20899-8920

**Keywords:** exponential backoff algorithm, medium access control, stability

## Abstract

Random access schemes for packet networks featuring distributed control require algorithms and protocols for resolving packet collisions that occur as the uncoordinated terminals contend for the channel. A widely used collision resolution protocol is the *exponential backoff* (EB). New analytical results for the stability of the (binary) EB are given. Previous studies on the stability of the (binary) EB have produced contradictory results instead of a consensus: some proved instability, others showed stability under certain conditions. In these studies, simplified and/or modified models of the backoff algorithm were used. In this paper, care is taken to use a model that reflects the actual behavior of backoff algorithms. We show that EB is stable under a throughput definition of stability; the throughput of the network converges to a non-zero constant as the offered load *N* goes to infinity. We also obtain the analytical expressions for the saturation throughput for a given number of nodes, *N*. The analysis considers the general case of EB with backoff factor *r*, where BEB is the special case with *r* = 2. We show that *r* = 1/(1 − e^−1^) is the optimum backoff factor that maximizes the throughput. The accuracy of the analysis is checked against simulation results.

## 1. Introduction

In a data network environment, many nodes of the network may share a communication medium for transmitting data packets, and the scheme of organizing and scheduling transmission of the data packets is called *medium access control* (MAC). Whether to have a centralized medium access control or to let the nodes contend for the medium access without central control is a network design decision determined by many factors. When the access control involves contention among nodes, the collision of packets transmitted by different nodes is possible; MAC schemes based on contention access require a random access scheduling method, such as a backoff algorithm, to schedule transmissions randomly in order to reduce the probability of collisions.

If multiple nodes transmit at the same time, a packet collision occurs and the nodes must reschedule the transmission of the packets. If the rescheduling algorithm is deterministic, the retransmissions also will collide, and the nodes will never be able to successfully transmit the packets. This situation can be avoided by a collision resolution protocol such as a backoff algorithm, where each node randomly selects a time slot within a given time interval—the “contention window”—in which to transmit its packet. This scheme reduces, but does not eliminate the probability of collision altogether. For given contention window, as the number of nodes with packets to transmit grows, the probability of collision also increases. If the contention window size is too small, many packet transmissions will experience collision and the retransmission of those packets will increase the effective load, aggravating the channel conditions. The *binary exponential backoff* (BEB), a widely used backoff algorithm, adjusts the contention window size by indirectly estimating the traffic in the communication medium at individual nodes, in effect by counting consecutive collisions involving the same packet. The node’s contention window size is doubled every time the packet experiences a collision and the contention window size is reset to its minimum value following a successful transmission [1]. BEB has been specified as part of the MAC protocol in several network standards, including the MAC layer of Ethernet local area networks (LANs) (IEEE 802.3) and the Distributed Coordination Function (DCF) of the wireless LAN standard IEEE 802.11 [2], [3].

In this paper, we assess the stability of BEB and analyze its performance using a model that closely resembles practical network transmission schemes and therefore is useful for system planning and analysis.

### 1.1 Stability of Backoff Algorithms

Many papers study the stability of backoff algorithms, including BEB, in terms of their effect on network performance as the offered load increases. However, these studies have produced contradictory results instead of a consensus: some prove instability, others show stability under certain conditions. The mixed results are due to differences in the analytical models and the definitions of stability used in the analysis.

Simplified and/or modified models of the backoff algorithm are often used to make analysis more tractable. However, simplification or modification can lead to very different analytical results. For example, Aldous [4] proved that BEB is *unstable* for an infinite-node model (a simplified model) for any non-zero arrival rate, while Goodman et al. [5] showed using a modified finite-node model that BEB is *stable* for sufficiently small arrival rates. Also, while modification of the model can make the analysis much simpler, the analytical result may have limited relevance because it cannot be guaranteed that the modified model exhibits the same behavior as the actual algorithm.

The various definitions of stability used in the studies of backoff algorithms can be classified into two groups. One group of studies uses a definition based on throughput and the other uses delay to define stability. Under the throughput definition, the algorithm is stable if the throughput does not collapse as the offered load goes to infinity [4] or is an increasing function of the offered load [6]. Under the delay definition, the protocol is stable if the waiting time is bounded. Systems that are stable under the delay definition can be characterized by bounded backlog of packets in the queue, or recurrent property of Markov chain [7].

Most of the analytical and simulation studies on stability treat BEB in the context of a specific network medium access control (MAC) protocol such as Ethernet etc. [8], [9], [10], [11], [12]. However, the characteristics of this protocol seem to have as much or more effect on the network performance results than the intrinsic behavior of BEB. Some of the analytical works that focus on BEB itself are summarized as follows:

Kelly and MacPhee [13] prove that for an acknowledgment based random access scheme there exists a critical arrival rate *ν*_c_, with the property that the number of packets successfully transmitted is finite with probability 0 or 1 according as *ν* < *ν*_c_ or *ν* > *ν*_c_. It is also shown that *ν*_c_ = 0 for any scheme with slower than exponential backoff, and *ν*_c_ = log 2 for BEB. They use an infinite-node model with Poisson arrivals, assuming that no node ever has more than one packet arrive at it. This result proves that BEB is unstable for *ν* > *ν*_c_, but leaves open the stability for *ν* < *ν*_c_.

In [4], Aldous shows that, with infinite-node and Poisson arrival assumptions, BEB is *unstable* in the sense that *N*(*t*)/*t* converges to zero as *t* goes to infinity for any non-zero arrival rate, where *N*(*t*) is the number of the successful transmissions made during the time [0,*t*]. This result solves the open problem left in [13], but the model Aldous uses is slightly different from Kelly and MacPhee’s model.

Goodman et al. prove in [5] that BEB is *stable* if the arrival rate of the system is sufficiently small in the sense that the backlog of packets awaiting transmission remains bounded in time. More specifically, they show that BEB is stable if the arrival rate is smaller than λ^*^(*n*), where λ^*^(*n*) ≥ 1/*n^α^* ^log^ *^n^* for some constant *α* and *n* is the number of nodes. They assume that each of the finite number of nodes *n* has a queue of infinite capacity.

In [14], Al-Ammal et al. give a tighter (greater) upper bound of the arrival rate than that given in [5] for the stability of BEB under the delay definition of stability. By using the same analytical model as in [5], they show that there is positive constant *α* such that, as long as *n* is sufficiently large and the system arrival rate is smaller than 1/*αn*^0.9^ then the system is stable for the *n*-user system. The upper bound in [14] is further improved in [15], where it is proved that BEB is stable for arrival rate smaller than 1/*αn*^1−^*^η^*, where *η* < 0.25. The main point of their work is that BEB is stable for an arrival rate that is inverse of a sublinear polynomial in *n*.

Finally, in [7], Håstad et al. show, using the same analytical model as in [5], that BEB is *unstable* whenever *λ_i_* ≥ *λ*/*n* for 1 ≤ *i* ≤ *n* and *λ* > 0.567 + 1/(4*n* − 2), or when *λ* > 0.5 and *n* is sufficiently large under the delay definition of stability, where *λ* is the system arrival rate and *λ_i_* is the arrival rate at node *i*.

In summary, these representative analyses indicate that BEB is unstable for an infinite-node model, and for a finite-node model it is stable if the system arrival rate is small enough but unstable if the arrival rate is too large. We note that they all assume slotted transmissions. While these analytical results are well established, because they are contradictory and do not represent the actual system, there remains doubt about the stability of BEB so that this question continues to be an open problem. As noted in [7] and [16], the infinite node model used in [13] and [4] is a mathematical abstraction with limited practical application, and except for [13], all of the studies cited actually analyze a modified version of BEB. For example, in BEB, after *i* consecutive packet transmission failures (collisions) a node selects for the next transmission *a single random slot* from the next 2*^i^* slots with equal probability, while in the modified versions after *i* collisions a node is assumed to transmit in each slot with probability 2^−^*^i^*. Clearly, it is easier to analyze such a modified version of BEB because of its memoryless nature, but it is not guaranteed that it has the same stability characteristics as BEB.

### 1.2 Approach of This Paper

In this paper, we analyze the stability of the original BEB algorithm by showing that the network throughput continues to be non-zero even when the number of nodes goes to infinity. The analysis considers the general case of exponential backoff (EB) with factor *r*; BEB is the special case with *r* = 2. In the notation of [4], we show that *N*(*t*)/*t* converges to a non-zero value as *t* goes to infinity, and we show that *p*_c_, the probability that a transmitted packet will experience a collision, is always smaller than 1/*r*.

Network performance measures are usually given as a function of the offered load. A commonly used definition of offered load is *N*, the number of nodes waiting to transmit; this concept underlies EB [8], which indirectly estimates the number of nodes contending by counting consecutive collisions. A second definition of offered load is the total packet arrival rate of the system, relative to the channel capacity. Since the purpose of EB is to alleviate the effects of contention among the nodes and to adapt the system to the number of nodes, the first definition of offered load is more appropriate for analyzing EB and is used in this paper. For the same reason, the performance of EB should be evaluated based on its effect on the measures of network efficiency, such as throughput.

In this paper, we assume a fixed number of nodes *N* in saturation condition. Here *saturation condition* means that each node always has a packet to transmit. Thus, *N* represents the offered load of the network. We also assume no errors on the channel. Under this assumption, we analyze network *throughput* for a slotted system with EB and compare the analytical results with simulation. The saturation condition assumption is also made by Bianchi in [17], where he used his own approach to analyze the throughput of the distributed coordination function (DCF) mode of the IEEE 802.11 wireless LAN standards.

This paper is organized as follows. In Sec. 2, we analyze EB to obtain the performance measures and establish stability. The analysis is carried out in several steps, which consists of modeling of EB, analysis of saturation throughput, and analysis of asymptotic behavior. Section 3 discusses some aspects of the simulation used to validate the analytical results. Section 4 is the conclusion of the paper.

## 2. Analysis of EB

In our analysis, the time is divided into time slots of equal length, and all packets are assumed to be of the same duration, equal to the slot time. Furthermore, all nodes are assumed to be synchronized so that every transmission starts at the beginning of a slot and ends before the next slot. At its first transmission, a packet is transmitted after waiting the number of slots randomly selected from {0, 1,⋯, *W*_0_ − 1}, where *W*_0_ ≥ 1 is an integer representing the minimum contention window size. Every time a node’s packet is involved in a collision, the contention window size for that node is multiplied by the *backoff factor r* and an integer random number *D_i_* is generated within the contention window for the next transmission attempt, where on a packet’s *i*-th retransmission

$$\mathrm{Pr}\left\{D_{i}=k,k=0,1,\cdots ,\left\lfloor r^{i}W_{0}\right\rfloor -1\right\}=\frac{X_{i}+1-Y_{i}}{X_{i}\left(X_{i}+1\right)}$$ (1)

$$\mathrm{Pr}\left\{D_{i}=\left\lfloor r_{i}W_{0}\right\rfloor \right\}=\frac{Y_{i}}{X_{i}+1}$$ (2)

where and $X_{i}=\left\lfloor r^{i}W_{0}\right\rfloor$ and *Y_i_* are the integer and fractional part of *r^i^W*_0_. *i* = 0 represents the first transmission attempt. For integer *r*, this operation is equivalent to randomly selecting a number from (0, 1, ⋯, *r^i^W*_0_ − 1) with equal probability 1/*r^i^W*_0_. With *r* = 2, this procedure is called *binary* exponential backoff.

### 2.1 Analytical Model of EB

The characteristic behavior of a backoff algorithm is critical when the channel is heavily loaded, and in fact, the very idea of EB is to cope with the heavily loaded channel condition. Thus, we analyze EB under saturation conditions. The saturation condition represents the largest possible load offered by the given number of nodes, which is a reasonable assumption for investigating EB.

**Fig. 1:**
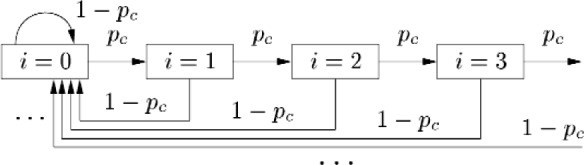
State transition diagram in a node for exponential backoff (EB).

We model the operation at an individual node using the state diagram in Fig. 1, in which each node is in one of an infinite number of backoff states and *p*_c_ denotes the probability that a transmission experiences a collision. In backoff state *i*, *i* = 0, 1, 2,⋯, the contention window size for a node is *W_i_* = *r^i^ W*_0_, where *W*_0_ is the minimum contention window size. As the diagram in Fig. 1 indicates, after a successful transmission, which occurs with probability 1 − *p*_c_, from any other state a node enters backoff state *i* = 0 with contention window size *W*_0_. While in backoff state *i* = *k*, after an unsuccessful transmission, a node enters backoff state *i* = *k* + 1 with probability *p*_c_.

Denote *B_k_* as the *k*-th state that a node enters. Then, *B_k_* is a Markov chain with the transition probabilities *p_i,j_*, *i*, *j* = 0, 1, ⋯, given as follows.

$$p_{i,0}=\mathrm{Pr}\left\{B_{k+1}=0|B_{k}=i\right\}=1-p_{c},$$ (3)

$$p_{i,i+1}=\mathrm{Pr}\left\{B_{k+1}=i+1|B_{k}=i\right\}=p_{c},$$ (4)

$$p_{i,j}=0,\;j\neq 0,\;j\neq i+1.$$ (5)

Let *P_i_* be a probability defined as

$$P_{i}=\underset{\;k\to \infty }{\lim}\mathrm{Pr}\left\{B_{k}=i\right\},\;i=0,1,\cdots ,$$ (6)

then *P_i_* is the relative frequency that a node will enter state *i* in the steady state. Since $\Sigma_{i=0}^{\infty }P_{i}=1$, from Fig. 1, *P_i_* can be obtained as follows

$$P_{i}=\left(1-p_{c}\right)p_{c}^{i},\;i=0,1,\cdots .$$ (7)

### 2.2 Throughput

The main performance measure in evaluating a network is its throughput. We analyze the saturation throughput by calculating the probability that there is a successful transmission in a time slot.

**Fig. 2:**
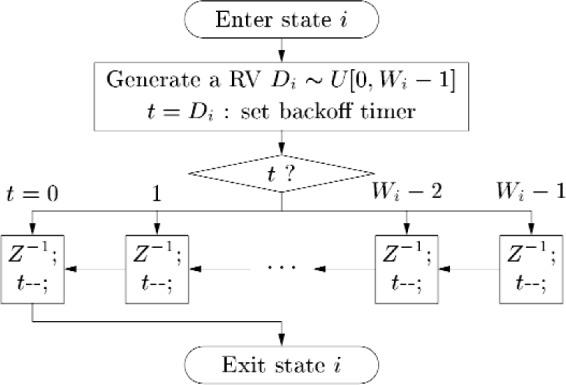
Procedure taken by a node in state *i*. *U*[0,*W_i_* − 1] represents a uniform distribution of integer over the interval [0,*W_i_*− 1], *Z*^−1^ means time delay by one time slot, and *t*-- is a decrement operation on the backoff timer.

The probability *P_i_* given in Eq. (7) is the relative frequency that a node *enters* state *i*. However, the average time a node *stays* in a state is different for each state and is a function of the contention window size of the state. As illustrated in Fig. 2, if a node enters state *i*, an integer random variable *D_i_* is generated according to Eqs. (1) and (2), and after waiting for *D_i_* time slots, the node will (re-)transmit the packet, after which the success or failure of the transmission will determine the next state of the node. Note that the node will stay in state *i* for *D_i_* + 1 time slots until the node moves to the next state. On average, a node will stay in state *i* for

$${\bar{d}}_{i}=E\left[D_{i}+1\right]=\frac{W_{i}+1}{2}$$ (8)

time slots. Let *S_i_* be the probability that a node is in state *i* at a given time; then *S_i_* specifies the distribution of nodes over the states. Since *S_i_* is proportional to $P_{i}{\bar{d}}_{i}$, it is given by

$$S_{i}=\frac{P_{i}{\bar{d}}_{i}}{\sum_{j=0}^{\infty }P_{j}{\bar{d}}_{j}}=\frac{\left(1-p_{c}\right)p_{c}^{i}\left(1-rp_{c}\right)\left(W_{i}+1\right)}{W_{0}\left(1-p_{c}\right)+1-rp_{c}},$$ (9)

where the summation in the denominator does not exist if *rp*_c_ ≥ 1. In fact, *rp*_c_ < 1 is a necessary condition for the system to reach steady state. Note that *S_i_* is given as a function of *p*_c_ and *W*_0_. Later, we show that the value of *p*_c_ is determined when the value of *W*_0_, and the number of nodes *N* are given.

Define Pr{*t* = *k* | *i*} as the conditional probability that a node’s backoff timer *t* will have value *k* given that the node is in state *i*. Since $\sum_{k=0}^{W_{i}-1}\mathrm{Pr}\left\{t=k|i\right\}=1$, it follows that

$$S_{i}=\sum_{k=0}^{W_{i}-1}S_{i}\cdot \mathrm{Pr}\left\{t=k|i\right\}=\sum_{k=0}^{W_{i}-1}s_{i,k},$$ (10)

where *s_i,k_* is the probability that the node is in state *i and* the backoff timer has value *k*. Since the backoff timer is decreased by one every slot time, *s_i,k_* satisfies

$$s_{i,k}=s_{i,W_{i}-1}\cdot \left(W_{i}-k\right),\;k=0,1,\cdots ,W_{i}-1.$$ (11)

By substituting Eq. (11) into Eq. (10), it can be shown that $s_{i,W_{i}-1}=S_{i}/\left({\bar{d}}_{i}W_{i}\right)$, and thus,

$$s_{i,k}=\frac{2\left(1-p_{c}\right)p_{c}^{i}\left(1-rp_{c}\right)}{W_{0}\left(1-p_{c}\right)+1-rp_{c}}\cdot \frac{W_{i}-k}{W_{i}}.$$ (12)

When *k* = 0, we have *s_i_*_,0_, the probability that a node is in state *i* and the backoff timer is expired, that is, a node will transmit a packet in state *i*.

Let *p*_t_ be the probability that a given node will transmit in an arbitrary time slot. Then, since *s_i_*_,0_, *i* = 0, 1, ⋯, are the probabilities of mutually exclusive events, $p_{t}=\sum_{i=0}^{\infty }s_{i,0}$. From Eq. (12)*s_i_*_,0_ = *s_i_*_−1,0_*p*_c_, *i* = 1, 2, ⋯. Thus,

$$p_{t}=\frac{s_{0,0}}{1-p_{c}}=\frac{2\left(1-rp_{c}\right)}{W_{0}\left(1-p_{c}\right)+1-rp_{c}}.$$ (13)

Note that *p*_t_ is a function of *p*_c_ and *W*_0_, but also related to *N* through the value of *p*_c_ as will be shown later. As we shall see in the following, since *p*_c_ goes to 1/*r* as *N* goes to infinity, *p*_t_ converges to zero as the number of nodes goes to infinity.

As noted in [17], the numerical value of *p*_t_ is also constrained by the fact that *p*_c_ can be expressed in terms of *p*_t_, that is

$$\begin{array}{l} p_{c}=1-\text{Pr}\left\{\text{none of the other}\;N-1\;\text{nodes transmits}\right\}\\ \;=1-{\left(1-p_{t}\right)}^{N-1}, \end{array}$$ (14)

where (1 − *p_t_*)*^N^* ^− 1^ is the probability that none of the other *N* − 1 nodes transmits. Solving Eq. (14) for *p*_t_, we have

$$p_{t}=1-{\left(1-p_{c}\right)}^{1/\left(N-1\right)}.$$ (15)

**Fig. 3:**
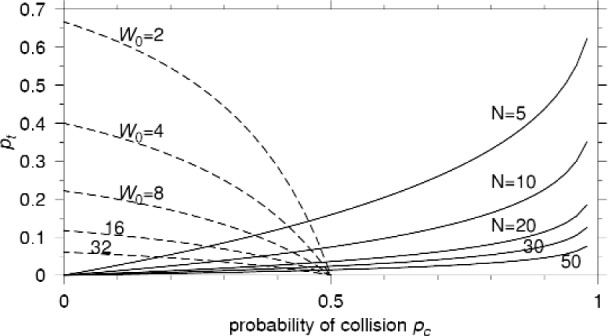
Plots of *p*_t_ as a function of *p*_c_; dashed lines: *p*_t_ in Eq. (13), solid lines: *p*_t_ in Eq. (15). *r* = 2.

**Fig. 4:**
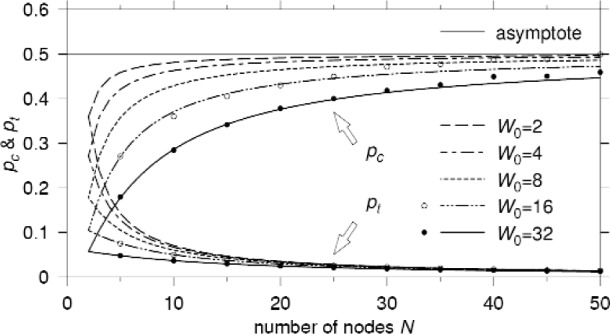
Plots of the probability of collision *p*_c_, and the probability of transmission *p*_t_. *r* = 2.

Since Eqs. (13) and (15) are two constraining equations for *p*_t_ as a function of *p*_c_, the unique intersection of these two equations gives us the values of *p*_c_ and *p*_t_ for given *N* and *W*_0_. Note that *p*_c_ is always less than 1/*r*, which is the requirement for the existence of the summation in Eq. (9). Figure 3 shows plots of *p*_t_ as a function of *p*_c_ given in Eq. (13) (dashed lines) for *r* = 2 and various values of *W*_0_, and in Eq. (15) (solid lines) for various values of *N*. The probability of collision *p*_c_ and the probability of transmission *p*_t_ obtained numerically from Eqs. (13) and (15) by calculating the intersection are plotted in Fig. 4. The plot shows *p*_c_ and *p*_t_ converging to 1/*r* (=0.5) and zero, respectively, as the number of nodes increases. The circles and bullets drawn along the curves are simulation results obtained for *W*_0_ = 16 and *W*_0_ = 32. Figure 4 shows that the analytical and simulation results agree extremely well. (More discussion on the simulation is given in Sec. 3.)

Since the channel is busy for a given time slot when there is at least one transmitting node in the slot time, the probability that the channel will be busy in a time slot is

$$P_{\text{busy}}=1-{\left(1-p_{t}\right)}^{N}=1-P_{\text{idle}},$$ (16)

where *P*_idle_ is the probability that a time slot is idle. On the other hand, a successful transmission occurs when there is only one transmitting node. Thus, the probability that there will be a successful transmission in a time slot is defined as

$$P_{\text{succ}}{=}_{N}C_{1}p_{t}{\left(1-p_{t}\right)}^{N-1}=Np_{t}{\left(1-p_{t}\right)}^{N-1},$$ (17)

where *_N_C*_1_ is the number of ways of choosing 1 out of *N* nodes. Note that a collision occurs if there are multiple nodes transmitting in the same time slot. Thus, the probability that a collision will occur in a time slot is given by

$$P_{\text{col}}=P_{\text{busy}}-P_{\text{succ}}.$$ (18)

**Fig. 5:**
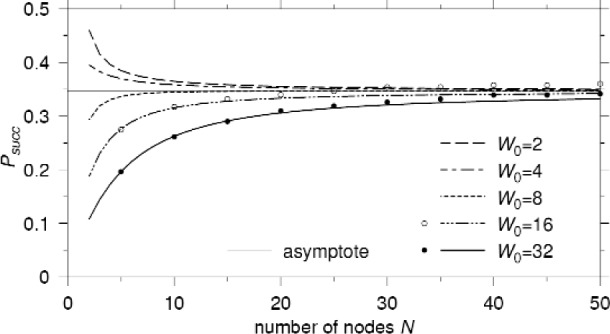
The probability of successful transmission in a slot (normalized saturation throughput). *r* = 2.

If we normalize the slot time as the unit time, in any given unit time duration, the average number of frames that are successfully transmitted is *P*_succ_. If we ignore the packet overhead, the normalized throughput is simply *P*_succ_. In the notation of [4], *P*_succ_ = lim*_t_*_→∞_ *N*(*t*)/*t*. Figure 5 shows plots of *P*_succ_ versus *N* for various values of *W*_0_. Note that *P*_succ_ converges to a non-zero constant ( $\frac{1}{2}\ln2$ to be precise when *r* = 2; see Eq. (25) in Corollary 2), which does not depend on *W*_0_, as the number of nodes increases. Even when there are a lot of nodes contending for the medium access, BEB manages to control the transmission attempts in a slot to guarantee sustained probability of successful transmission. In fact, it is shown below in Sec. 2.3 that the average number of nodes that transmit in a slot converges to a constant less than 1 as the number of nodes goes to infinity. Note that, for a small number of nodes and large *W*_0_, *P*_succ_ increases as the number of nodes increases. This behavior occurs because of the large number of idle time slots on the channel, and increasing the number of nodes increases the efficiency of the channel usage leading to a higher *P*_succ_.

### 2.3 Stability and Asymptotic Behavior of EB

Now we investigate the asymptotic behavior of EB observed when the number of nodes *N* goes to infinity. As shown in Fig. 4, *p*_t_ converges to zero as the number of nodes increases, due to the increased contention window sizes which causes a smaller probability of transmission in a given time slot. The following theorem describes the asymptotic behavior of *p*_t_.

*Theorem 1*: Define *n*_t_ = *N* · *p*_t_ as the expected number of nodes that will transmit in an arbitrary time slot. Then, *n*_t_ converges to the non-zero value ln[*r*/(*r* − 1)] as the number of nodes *N* goes to infinity.

Proof We first show that *p*_c_ converges to 1/*r*, and *p*_t_ converges to zero as *N* goes to infinity. From Eqs. (13) and (15), we have

$$1-{\left(1-p_{c}\right)}^{\frac{1}{N-1}}=\frac{2\left(1-rp_{c}\right)}{W_{0}\left(1-p_{c}\right)+1-rp_{c}}.$$ (19)

Take an infinite limit of *N* on both sides, then

$$\begin{array}{l} \underset{N\to \infty }{\lim}\left(1-{\left(1-p_{c}\right)}^{\frac{1}{N-1}}\right)=\underset{N\to \infty }{\lim}\frac{2\left(1-rp_{c}\right)}{W_{0}\left(1-p_{c}\right)+1-rp_{c}},\\ \;=0 \end{array}$$

which implies lim*_N_*_→∞_ 2(1 − *rp*_c_) = 0. Thus,

$$\underset{N\to \infty }{\lim}p_{c}=\frac{1}{r},\;\underset{N\to \infty }{\lim}p_{t}=0.$$ (20)

Now, rewrite Eq. (13) to yield *p*_c_ as a function of *p*_t_ as follows:

$$p_{c}=\frac{2-\left(1+W_{0}\right)p_{t}}{2r-\left(r+W_{0}\right)p_{t}}$$ (21)

By equating Eqs. (14) and (21), we have

$$\frac{r-1}{r}\cdot \frac{2-p_{t}}{2-\left(1+\frac{W_{0}}{r}\right)p_{t}}={\left(1-p_{t}\right)}^{N-1}.$$ (22)

Since lim*_N_*_→∞_ *p*_t_ = 0, by multiplying both sides by 1 − *p*_t_ and taking the limit as *N*→∞, we have

$$\frac{r-1}{r}=\underset{N\to \infty }{\lim}{\left(1-p_{t}\right)}^{N}.$$ (23)

By taking natural logarithm of both sides, Eq. (23) can be written as

$$\begin{array}{l} \ln\frac{r-1}{r}=\underset{N\to \infty }{\lim}N\ln\left(1-p_{t}\right)\\ \;=\underset{N\to \infty }{\lim}Np_{t}\underset{N\to \infty }{\lim}\frac{\ln\left(1-p_{t}\right)}{p_{t}}\\ \;=-\underset{N\to \infty }{\lim}Np_{t}, \end{array}$$

which concludes the proof. □

This theorem tells us two very important facts. First, *n*_t_ converges to a finite positive constant. In fact, with *r* = 2, *n*_t_ converges to ln 2 < 1. Thus, no matter how many nodes the network contains, it can be expected that, on average, less than one node will try to transmit in any time slot, which in turn guarantees non-zero throughput of the network regardless of the number of the nodes in the network as shown in the following corollary. Secondly, lim*_N_*_→∞_ *n*_t_ is not a function of *W*_0_. Thus, the minimum contention window size does not affect the asymptotic behavior of the network.

**Fig. 6:**
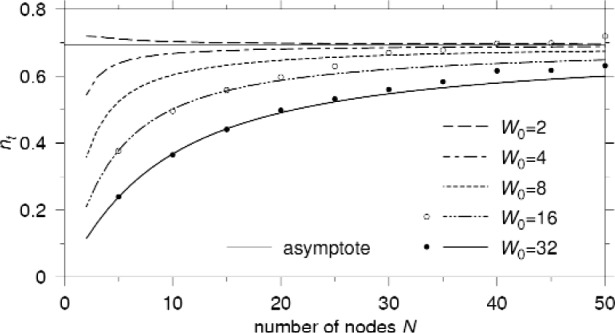
The expected number of nodes *n*_t_ that will transmit in an arbitrary time slot. *r* = 2.

Fig. 6 shows the plots of *n*_t_ vs *N* along with simulation results. With a larger minimum contention window size *W*_0_, the expected number of transmitting nodes in a slot is smaller because of the longer average backoff by each node. But as the number of nodes increases, all curves converge to the asymptote lim*_N_*_→∞_ *n*_t_ = ln 2 for *r* = 2, which is shown with a thin line in Fig. 6.

*Corollary 2*: The probability *P*_busy_ that channel is busy, and the probability *P*_succ_ that there will be a successful transmission in a time slot converge as the number of nodes *N* goes to infinity as follows:

$$\underset{N\to \infty }{\lim}P_{\text{busy}}=\frac{1}{r},$$ (24)

$$\underset{N\to \infty }{\lim}P_{\text{succ}}=\frac{r-1}{r}\ln\frac{r}{r-1}.$$ (25)

The proof of Corollary 2 is straightforward from Theorem 1. Note that the limits in Eqs. (24) and (25) do *not* depend on *W*_0_ and are functions of only *r*, the backoff factor. Equation (24) shows that, even with a large number of nodes, the channel is idle about 50 % of the time (for *r* = 2), which guarantees sustained non-zero probability of successful transmission. This is due to the backoff mechanism controlling transmission attempts by the nodes. As noted in Sec. 2.2, *P*_succ_ represents the normalized throughput. Thus, the asymptote of *P*_succ_ in Eq. (25), drawn in Fig. 5 with thin solid line, shows that EB is stable under the throughput definition.

As shown in Eq. (25), the throughput in the limit (the asymptotic throughput) is a function of the backoff factor *r*. The optimum backoff factor that maximizes the asymptotic throughput is

$$r_{\text{opt}}=\frac{1}{1-e^{-1}}$$ (26)

which yields the maximum asymptotic throughput

$${\left(\underset{N\to \infty }{\lim}P_{\text{succ}}\right)}_{\text{opt}}=\frac{1}{e}\approx 0.368.$$ (27)

**Fig. 7:**
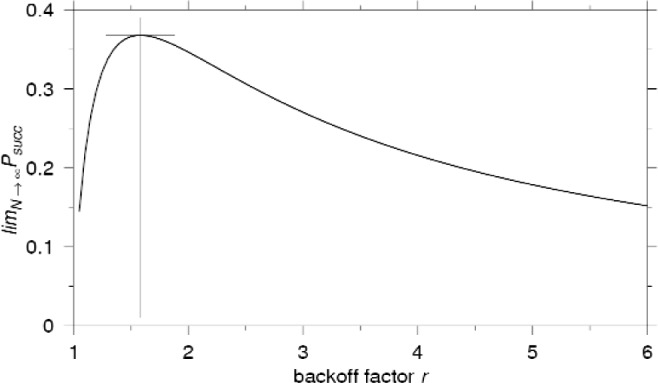
The optimum backoff factor *r*.

Fig. 7 shows the plot of $\underset{N\to \infty }{\lim}P_{\text{succ}}$ vs *r*. Note that the *binary* exponential backoff (*r* = 2) produces the asymptotic throughput $\frac{1}{2}\text{ln}\;2\approx 0.347$ which is quite close to the asymptotic throughput of the optimum EB.

## 3. Simulation

To support our analysis, simulation results are added in Figs. 4–6, which are represented by circles and bullets, along with the curves of analytical results. The simulator is written in the C++ programming language, and simulation results were obtained by running 500 000 time slots after 10 000 time slots of warming up for *W*_0_ = 16, 32, and *N* = 5, 10, ⋯, 50.

The simulation results in Figs. 4–6 agree with those obtained from our analysis. However, when there are relatively many nodes, slight differences between the analytical and simulation results are observed which can be attributed to a *starvation* effect. A starvation effect is different from a capture effect. A capture effect makes only a few nodes consume the whole transmission channel, but a starvation effect gives a few nodes little chance to transmit their packets while most of nodes have fairly good chances. For example, for *N* = 50 and *W*_0_ = 16, while most nodes tried between 7000 and 8000 packet transmissions during 500 000 time slots, there was a node with only five transmission tries. Also, there were several nodes with less than 3000 transmission attempts. For smaller *W*_0_, however, a capture effect [18] was observed instead of starvation, whose result is not included in the figures. The reason for these effects is the necessarily finite observation time of the simulation.

## 4. Conclusion

We analyzed the performance of EB to obtain the saturation throughput. The stability of EB was also established by showing the asymptotic behavior of EB when the number of nodes goes to infinity. From the analysis results, we showed that EB guarantees a certain amount of throughput no matter how many nodes are present in the network. We also showed that the optimum backoff factor for EB which maximizes the asymptotic throughput is *r* = 1/(1 − e^−1^).

In this paper, a new and efficient analytical method was used to analyze the characteristics of EB. This analytical method can also be applied to analyze network protocols using EB, such as IEEE 802.11 DCF.
